# Supplementary material for: Photobiomodulation therapy in improvement of harmful neural plasticity in sodium salicylate-induced tinnitus
Source: PLoS One. 2024 Apr 16;19(4):e0296607. doi: 10.1371/journal.pone.0296607 (PMC11020422; doi:10.1371/journal.pone.0296607)
Supplement: Attachment — Submitted filename: response to reviewers.docx [file pone.0296607.s001.docx]

Dear reviewers,

Greetings!

We appreciate your valuable comments and are grateful that these comments have improved the article. We have implemented all your comments and uploaded all figures in better quality, as shown in the track change file.

Reviewer#1

- Line 57 perception of sound THAT occurs √ Done
- Line 72 "needed for widespread use" needs to be explained more clearly√ the paragraph was changed
- The paragraph from line 78 is very clumsy: √the paragraph was changed
- Line 99 "In scientific sources" is better expressed as "PREVIOUS WORK HAS SHOWN"√
- Line 128 "In animal models, tinnitus IS USUALLY INDUCED by"√
- Line 114 the sentence beginning with "Then, during ...." appears incomplete or clumsy √ the sentence was changed
- Line 188 is "should be" the correct way to express this√
- Line 358 replace "common, complex and cure-less symptom" with something like "common AND complex CONDITION THAT HAS NO CURE"√ Was replaced
- Line 356 I am not sure that "The GIN value represented the percentage inhibition", rather that the GIN ratio was calculated by ...? √ Done
- Line 379 & 382 replace "in the review articles" with "in PREVIOUS STUDIES"√ Done
- Line 382 replace not confirmed with NOT DEMONSTRATED√ Done
- Line 441 replace "needed to be confirmed " with "NEEDED TO CONFIRM THIS"√ Done

- In the methods section, generally the study design is followed by the treatments (anesthesia etc., PBMT) followed by outcome measures, and lastly followed by statistics and other analyses. √ relocation done
- The table of PBMT parameters lists energy density as 99J/cm2. This is at the optical fiber outlet? Was there any effort to measure the energy density that the animal actually received? This needs to be addressed√ it was addressed
- I think that the last paragraph of the Discussion beginning line on 449 would fit better as the concluding paragraph of the Conclusion√ *Relocation done*

Reviewer # 2

-The introduction is too long. The information on plasticity could be reduced. √ *Done*

- Also, information on the method for confirming the occurrence of tinnitus could be reported in the methodology, or in the discussion, to clarify the findings with the model used. √ *Relocation done*
- The anesthesia topic is before the photobiomodulation topic. Please provide the information in chronological order in your methodology. In addition, your methodology should end with the topic of statistical analysis. √ *Done*
- Figure 1 does not have a good resolution. Please include a figure with a good resolution. √ *Done*
Did the animals in the control group receive the placebo treatment? If so, include this information in the methodology. How was this procedure carried out? √ *It was explained that we did not have placebo group.*
- Maintain in the graphs only the symbols that represent a significant difference. Remove the bars with the abbreviation “ns”. *We did not just remove the ns symbols from the graph bars because the non-significance of the difference between the GIN value of the PBMT group compared to the control group indicates the response to treatment, which is very important in this study and we wanted to highlight it. In addition, the non-significance of the difference between PPI values among groups which indicates the normal hearing is also important in our study. However, please let us know if you insist that these marks be removed and we will respectfully remove all ns marks.*

- In addition, the resolution of the graphs needs to be improved. √ *Done*
- I suggest that the Electrophysiological (ABR) test topic be before the GPIAS test. This seems to be more consistent with your experimental design. √ *Done*
- Include the ANOVA value in the description of the results. If so, you could use a description that indicates the observed phenomenon more precisely. For example: Animals from tinnitus group showed the reduction of the mean GIN value, compared to the control group. However, Animals from photobiomodulation group showed an increase of the mean GIN value, compared to the control group. √ *Done*
When describing the results, clearly present the ANOVA values and the post hoc values√ *Done*
The figures need a better resolution√ *Done*
Insert the topic limitations of the study at the end of the discussion. √ *Done*
